# We Are All in This Together: COVID-19 and a Call to Action for Mental Health of Children and Adolescents

**DOI:** 10.3389/fpsyt.2020.589834

**Published:** 2021-02-11

**Authors:** Priscilla Idele, Prerna Banati

**Affiliations:** ^1^UNICEF Office of Research at Innocenti, Florence, Italy; ^2^UNICEF West and Central Africa Regional Office, Dakar, Senegal

**Keywords:** mental health, COVID-19, children, adolescent, psychosocial

The COVID-19 pandemic has exposed the urgent need to tackle the crisis of mental health among children and young people. We call for a multi-stakeholder Global Mental Health Alliance for Children, which would achieve the following objectives: to strengthen evidence and understanding of mental health and well-being, causes and risks for children and young people; to scale up investment in mental health programming for children and young people, and particularly expanding the global cadre of health workers, social workers and community workers, with a focus on prevention and promotion of mental health; to support youth-led, evidence- and rights-based initiatives; to expand advocacy and knowledge of mental health for children and young people among the wider public, and reduce stigma, marginalization and discrimination against those experiencing mental ill-health; and to enhance funding from both the public and private sectors for promotion of mental health, prevention of ill-health and treatment of mental health disorders.

For children already living with mental ill-health, the COVID-19 pandemic is exacerbating an already grave and growing challenge. Before the pandemic, between 10 and 20 per cent of all children and adolescents were estimated to suffer from some type of mental health disorder, and mental health conditions accounted for around 16 per cent of the global burden of disease and injury among adolescents (1). Worldwide, depression is estimated to be among the leading causes of disability among young people and suicides are the third leading cause of death among adolescents, and the second among 15–19-year-old adolescent girls. Fifty percent of mental health conditions arise before the age of 14, and 75 per cent by the mid-20s (1, 2).

Recent literature suggests elevated mental health impacts of COVID-19 on children and adolescents (3–5). A study of 8,079 students aged 12–18 years reported prevalence of depressive and anxiety symptoms (identified using Patient Health Questionnaire (PHQ-9) and the Generalized Anxiety Disorder (GAD-7) questionnaire at 43.7 and 37.4%, respectively, with female students at elevated risks (5). These staggeringly high figures become even more worrying given the formative nature of childhood and adolescence. Developmental science evidence has demonstrated the lifetime and intergenerational consequences of exposures to mental stressors early in life. Wemakor and Mensah (6) found that children of depressed mothers 15- 45 years old in northern Ghana were three times more likely to be stunted, noting that younger mothers were more likely to be depressed. Sherr (7) describes the consequences of adverse childhood impacts on mental health in later life, noting the well-documented consequences, including physical illness, death, divorce and unemployment. The risks to future human capital are significant and threaten economic growth and development particularly for nations seeking to reap demographic dividends.

Unfortunately, COVID-19 is not the first major global threat to impact mental well-being of children and adolescents. Various health, economic, societal, environmental and developmental risks have rocked the world and impacted the mental and psychosocial well-being of the youngest generation. Evidence demonstrates the impacts of the global financial crisis on youth suicide rates, hitting men 15–24 hardest (8), the East Asian tsunami disaster resulted in chronic Post Traumatic Stress Disorders (PTSD) (9), exposure of children to armed conflict (10), and disease shocks such as HIV (11) have all seen impacts on increased anxiety and stress.

In an increasingly globalized world, the risks to children from such shocks are magnified. If the COVID-19 crisis has taught us anything, it is that global development partners, governments, communities, caretakers and young people themselves can no longer simply react to global shocks but must put in place measures to anticipate the most plausible risks and build resilience measures and contingency plans to protect the mental health of children and adolescents at the global, national, societal and individual levels.

To do so, the wide evidence gulf needs to be addressed, including assessing the burden in all contexts, understanding vulnerabilities and impacts, unpacking the social, environmental and health determinants of mental health and well-being, identifying solutions to fight stigma and discrimination, and looking critically at quality, durability, sustainability, and coverage of interventions, especially psychotherapy.

However, the absence of evidence is not evidence of absence. Some countries have taken on board mental health as a national issue. Significant political commitment by Sri Lanka after the 2004 Tsunami led to dedicated financing, efforts to decentralize mental health services and integration of mental health into primary health care across the island (12). The need for sustained political commitment and will, adequate national financing and development assistance, infrastructure and systems, and enduring inclusive governance mechanisms and concerted action by all partners is imperative. Vigo et al. (13) call for a partnership bringing together global and country level stakeholders including civil society, donors and development agencies.

Many lessons can be learnt from responses to the AIDS pandemic [see (14), this volume]. Notably, vocal national leadership and the institutionalization of a national response through inclusive National AIDS councils and bold accountability frameworks helped to accelerate action and progress in reaching the most vulnerable.

To respond across the spectrum of promotion—prevention—treatment and care—a coordinated multisectoral approach is imperative. Mental health is not a health sector issue alone. The role of the education sector is central to delivering and sustaining interventions, with evidence that whole-school multicomponent interventions can improve psychosocial outcomes for children (15, 16). Social welfare workforce and child protection services are often at the frontline, mitigating and reporting abuse and adverse childhood experiences. The health sector is critical, through systems level responses (including augmentation of global mental health workforce) as well as at the community level (through peer and lay-counselors) and links to primary health care.

Over the last decades, the global community has invested heavily in global partnerships to tackle development crises through effective financing of solutions. Multi-stakeholder Global Health Partnerships such as the Global Fund to fight AIDS TB and Malaria and the GAVI Alliance for vaccines and immunizations are effective and sustainable models with many positive attributes to build on, including taking a whole-of-society approach, promoting national ownership, engaging with affected populations, and leveraging new partners including the private sector (17). We are all in this together which means rallying together for impact across UN agencies, national governments, private sector, community-based organizations, academia and centrally, with young people themselves.

## Author Contributions

All authors listed have made an equal, substantial, direct and intellectual contribution to the work, and approved it for publication.

## Conflict of Interest

The authors declare that the research was conducted in the absence of any commercial or financial relationships that could be construed as a potential conflict of interest.
